# Mechanical interplay between invadopodia and the nucleus in cultured cancer cells

**DOI:** 10.1038/srep09466

**Published:** 2015-03-30

**Authors:** Or-Yam Revach, Allon Weiner, Katya Rechav, Ilana Sabanay, Ariel Livne, Benjamin Geiger

**Affiliations:** 1 Department of Molecular Cell Biology, Weizmann Institute of Science, Rehovot 7610001, Israel; 2 Department of Materials and Interfaces, Weizmann Institute of Science, Rehovot 7610001, Israel; 3 Department of Chemical Research Support, Weizmann Institute of Science, Rehovot 7610001, Israel

## Abstract

Invadopodia are actin-rich membrane protrusions through which cells adhere to the extracellular matrix and degrade it. In this study, we explored the mechanical interactions of invadopodia in melanoma cells, using a combination of correlative light and electron microscopy. We show here that the core actin bundle of most invadopodia interacts with integrin-containing matrix adhesions at its basal end, extends through a microtubule-rich cytoplasm, and at its apical end, interacts with the nuclear envelope and indents it. Abolishment of invadopodia by microtubules or src inhibitors leads to the disappearance of these nuclear indentations. Based on the indentation profile and the viscoelastic properties of the nucleus, the force applied by invadopodia is estimated to be in the nanoNewton range. We further show that knockdown of the LINC complex components nesprin 2 or SUN1 leads to a substantial increase in the prominence of the adhesion domains at the opposite end of the invadopodia. We discuss this unexpected, long-range mechanical interplay between the apical and basal domains of invadopodia, and its possible involvement in the penetration of invadopodia into the matrix.

Invadopodia are actin-rich protrusions of the plasma membrane, which play a key role in the proteolytic degradation of the extracellular matrix (ECM)^1^,^2^,^3^,^4^. They are commonly found in cancer cells, where they are believed to drive cell invasion into the surrounding connective tissue and, consequently, promote the dissemination of metastases^5^,^6^,^7^. Correlative light and transmission electron microscopy (TEM) have demonstrated that invadopodia are membrane protrusions found mostly in close proximity to the nucleus and the Golgi system^8^,^9^,^10^.

The formation of invadopodia and their turnover are regulated by multiple external and cellular mechanisms^1^,^2^,^4^,^6^. Their key structural component is an actin bundle, the polymerization of which is regulated by nucleating proteins such as cortactin and the arp2/3 complex^7^,^11^,^12^,^13^. Another important protein that regulates invadopodia is the scaffold protein TKS5^14^,^15^ which, following phosphorylation by src-family kinases, associates with and drives the assembly of invadopodia through its interactions with NCK^15^,^16^ and N-WASP^17^. Suppression of TKS5 expression or inhibition of src-mediated phosphorylation leads to the disassembly of invadopodia, and loss of matrix degradation^18^,^19^. Microtubules were also shown to play an essential role in the formation and maintenance of invadopodia: their disruption by nocodazole blocks matrix degradation^20^, invadopodia elongation, and maturation^21^,^22^.

The protrusive activity of invadopodia is achieved by a combination of local adhesion to the matrix via integrins and associated proteins^23^,^24^, local enzymatic degradation of the matrix^2^,^5^,^6^,^10^,^13^, and physical force, generated by actin polymerization in the invadopod core^1^,^13^,^25^,^26^,^27^. It was previously suggested that unlike podosomes, which contain a distinct adhesive domain, invadopodia of cancer cells lack an adhesive capacity^5^,^6^. More recently, vinculin, paxillin and Hic-5 were detected in rings located at the periphery of newly formed invadopodia^23^,^24^. Blocking of integrin-mediated adhesion resulted in a reduction of matrix degradation^23^. Apparently, tight spatial and temporal coordination between adhesion, degradation, and actin polymerization-mediated pushing is needed for effective penetration of invadopodia into the ECM^27^; yet how all these mechanical elements are integrated at the “systems level” is still unknown.

In this study, we explored the mechanical interplay between the basal aspect of the invadopod's actin core, pointing towards the integrin adhesions, and the apical aspect, directed towards the nucleus. To obtain high-resolution 3D views of invadopodia, we developed a novel correlative microscopy approach that enables reconstruction of invadopodia and associated cellular structures, using a cultured A375 metastatic melanoma cell line as our main model system. These studies demonstrated that invadopodia are tightly packed, actin-based, and organelle-free cylindrical protrusions that span the space between the ventral cell membrane and the nucleus, extending through a dense web of microtubules. Immunolabeling for integrins and associated adhesome components indicated that invadopodia associate transiently with an adhesion ring containing integrins and cytoplasmic adhesome components.

Strikingly, the “apical tips” of >80% of the actin core bundles of invadopodia co-localized with conspicuous, 400–500 nm deep indentations in the nuclear membrane. Monitoring these nuclear indentations in live cells using total internal reflection fluorescence (TIRF) microscopy indicated that disassembly of invadopodia, induced by src or microtubule inhibitors, results in the loss of these indentations. Washout of the inhibitors leads to formation of new invadopodia and new corresponding nuclear indentations. Calculations of the mechanical force needed to induce the observed nuclear deformation suggest that the “pushing force” of an individual invadopod falls within the range of a few nanoNewtons. Interestingly, knockdown of the LINC complex components nesprin 2 or SUN1, an actin-binding nuclear envelope complex^12^,^28^,^29^,^30^, enhanced the prominence of ECM adhesions around invadopodia, suggesting that molecular interactions at the invadopod's apical tip regulate the interaction with the adhesive domain in the basal region. Taken together, these findings reveal multiple mechanical interactions between the actin cores of invadopodia and specific cellular structures, including the cell nucleus, the microtubular network, and integrin-mediated ECM adhesions, which may collectively contribute to the invasive phenotype of the cells.

## Results

### Invadopodia are transiently associated with adhesion rings at their basal aspect

**Figure 1:**
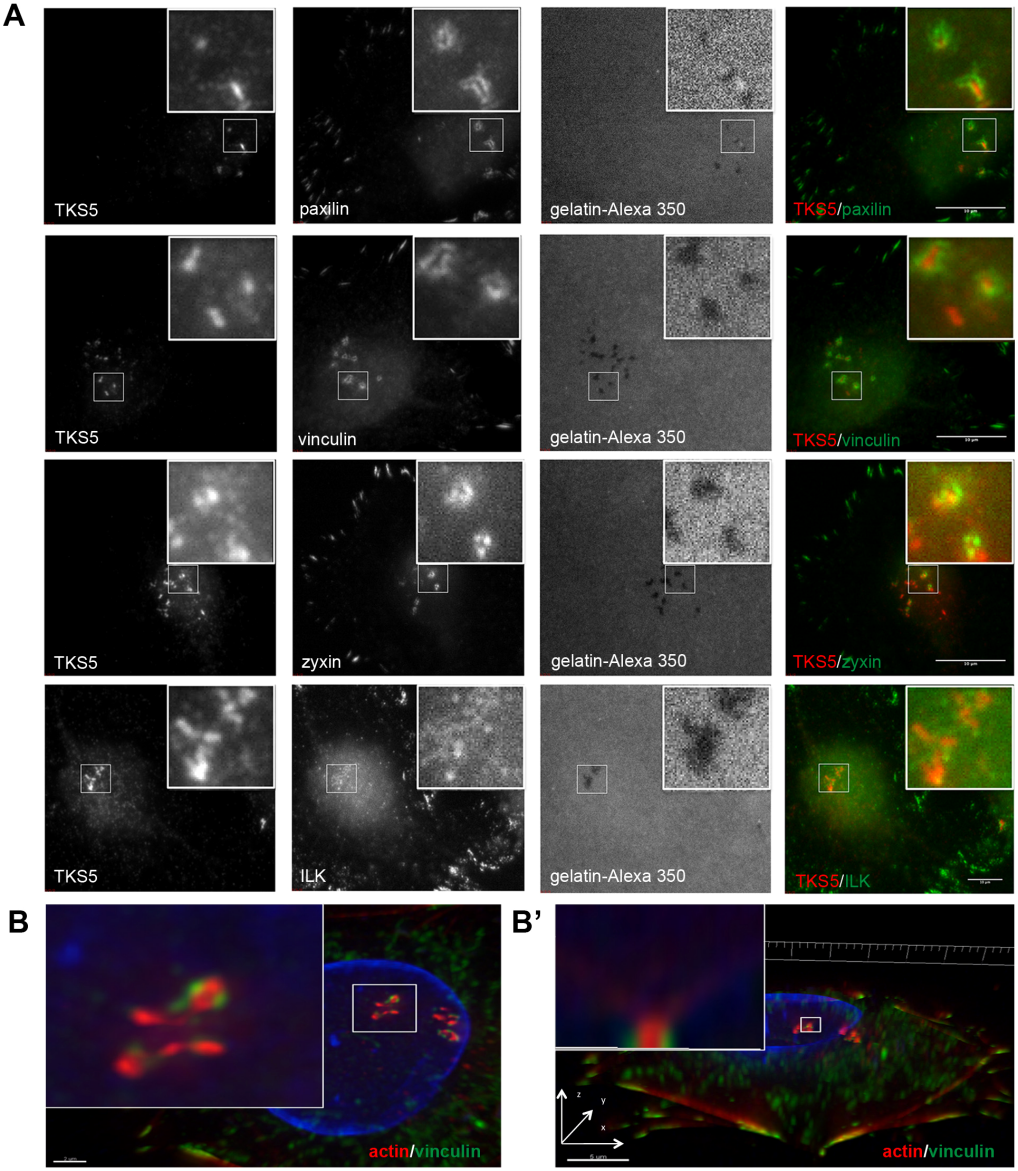
Invadopodia are associated with the ECM at their ventral aspect via integrin-mediated adhesion rings. (A) A375 cells were cultured overnight on gelatin-Alexa 350-coated coverslips with MMP inhibitor. ECM adhesions formed 1 or 2 h after inhibitor withdrawal. Cells were fixed and co-stained for TKS5 (red) and either vinculin, paxillin, zyxin or ILK (green). Invadopodia are enlarged within the white frames. Scale bar = 10 μm. (B) Three-dimensional deconvolution of invadopodia formed by A375, cultured for 2 h on gelatin-coated coverslips. Cells were fixed and co-stained for actin (red) and vinculin (green). Cells were kept in PBS, Z stack images were acquired, and 3D reconstruction was carried out using Imaris software. In B′, a side view of the marked invadopod is shown showing the association of vinculin with the ventral aspect of the actin bundle.

Preliminary attempts to localize different components of integrin-mediated adhesions in invadopodia pointed to considerable variability in their organization. To systematically characterize the assembly of adhesion plaques in invadopodia, A375 melanoma cells were cultured on fluorescently tagged gelatin in the presence of 25 μM GM6001 MMP inhibitor. Following an overnight spreading period, the inhibitor was washed away, and replaced with complete culture medium. Cells were fixed at different time points, and stained for the invadopodial markers TKS5 or actin, in conjunction with different adhesome proteins (β1 and β3 integrins, vinculin, paxillin, zyxin, or ILK). Within 1 hour after inhibitor withdrawal, a dot-like actin/TKS5 core was surrounded by ring-like adhesions. This association of the actin/TKS5 core with the adhesome components was, however, transient, since upon longer incubation (e.g., 6 h), the labeling intensity of the adhesion rings decreased significantly (Supplementary Fig. 1). The transient interaction of invadopodia with adhesion rings was also monitored by live-cell time-lapse microscopy, with or without pretreatment with MMP inhibitor (Supplementary Movie 1). Careful examination of A375 adhesion rings showed that vinculin and paxillin were predominantly associated with uniform rings surrounding the TKS5-rich core (Fig. 1A, Lanes 1 and 2), in agreement with previous reports^23^, whereas zyxin displayed a “bead-on-a-string” appearance (Fig. 1A, Lane 3), and ILK accumulated around the core in small dots, and displayed a scattered appearance (Fig. 1A, Lane 4). Integrins β1 and β3 also localized to the rings as dispersed dots around the core (Supplementary Fig. 2A, 2B). This organization suggests that the adhesion ring contains sub-domains of varying shape and molecular composition. To determine whether these characteristics of invadopodia-associated adhesion rings are general or A375-specific, we repeated the experiments, using vinculin labeling of MDA-231 metastatic breast cancer cells (Supplementary Fig. 2C).

Additional information concerning the differential 3D distributions of the actin bundle and the adhesion ring was obtained by 3D deconvolution fluorescence microscopy. As shown (Fig. 1B, B′), we were able to obtain “side views” of invadopodia, showing that the actin core extends into the cytoplasm, away from the ventral plasma membrane, whereas the vinculin ring is restricted to the membrane's focal plane.

### Reconstruction of the three-dimensional structure of invadopodia, based on correlated light and electron microscopy

To further elucidate the overall 3D organization of invadopodia, and visualize their diverse cellular interactions, we developed a correlated light and electron microscopy technique, enabling the imaging of invadopodia (identified by fluorescence microscopy) using a focus ion beam-scanning electron microscopy (FIB-SEM) “slice-and-view” approach. Unequivocal identification of invadopodia using a specific molecular marker is essential for such studies, given that invadopodia display structural heterogeneity, and are not found in every cell. Using this method, we could image the 3D spatial relationships of the invadopod core with different cellular compartments, including the ventral plasma membrane, the surrounding cytoskeleton, and the nucleus.

**Figure 2:**
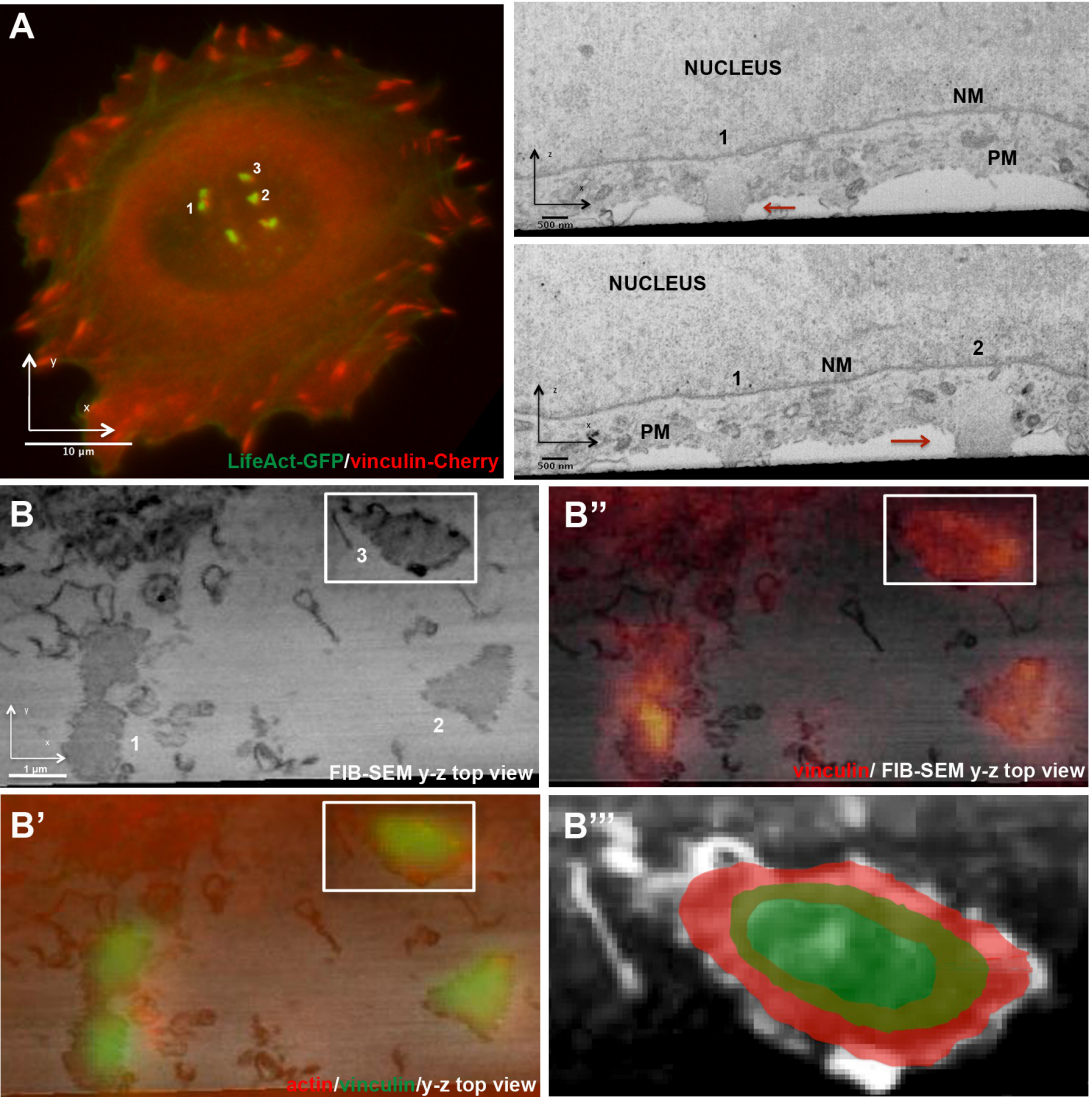
Three-dimensional imaging of invadopodia, using correlative light-FIB-SEM microcopy. (A) A375 cells co-expressing LifeAct-GFP (green) and mCherry vinculin (red) were plated on gelatin gel-coated gridded coverslips, and cultured for 2 h (see Materials and Methods). Left panel: Invadopodia as imaged by fluorescence microscopy, using a 100x/1.3 oil objective. Invadopodia #1–3 were then identified in the FIB-SEM and imaged using the “slice and view” mode (10 nm-thick slices; total thickness of the imaged area: 10 μm). Right panel: Two slices viewed by FIB-SEM. Invadopodia that correspond to the fluorescence image are denoted by the numbers 1 and 2, and by red arrows. NM: nuclear membrane; PM: plasma membrane. (B) Reconstruction of the “top-down view” obtained by the FIB-SEM and showing the slice closest to the matrix (invadopodia marked 1, 2, and 3). (B′) The same FIB-SEM top-down view is shown, overlaid with actin (red) and vinculin (green). (B″) The same FIB-SEM top-down view, overlaid with vinculin (red), showing the adhesion ring surrounding the invadopod core. (B′″) Magnification of invadopod #3′ (as in “B”, shown here in inverted contrast), superimposed on a schematic representation of vinculin (red ring) and F-actin (green) immunofluorescence.

**Figure 3:**
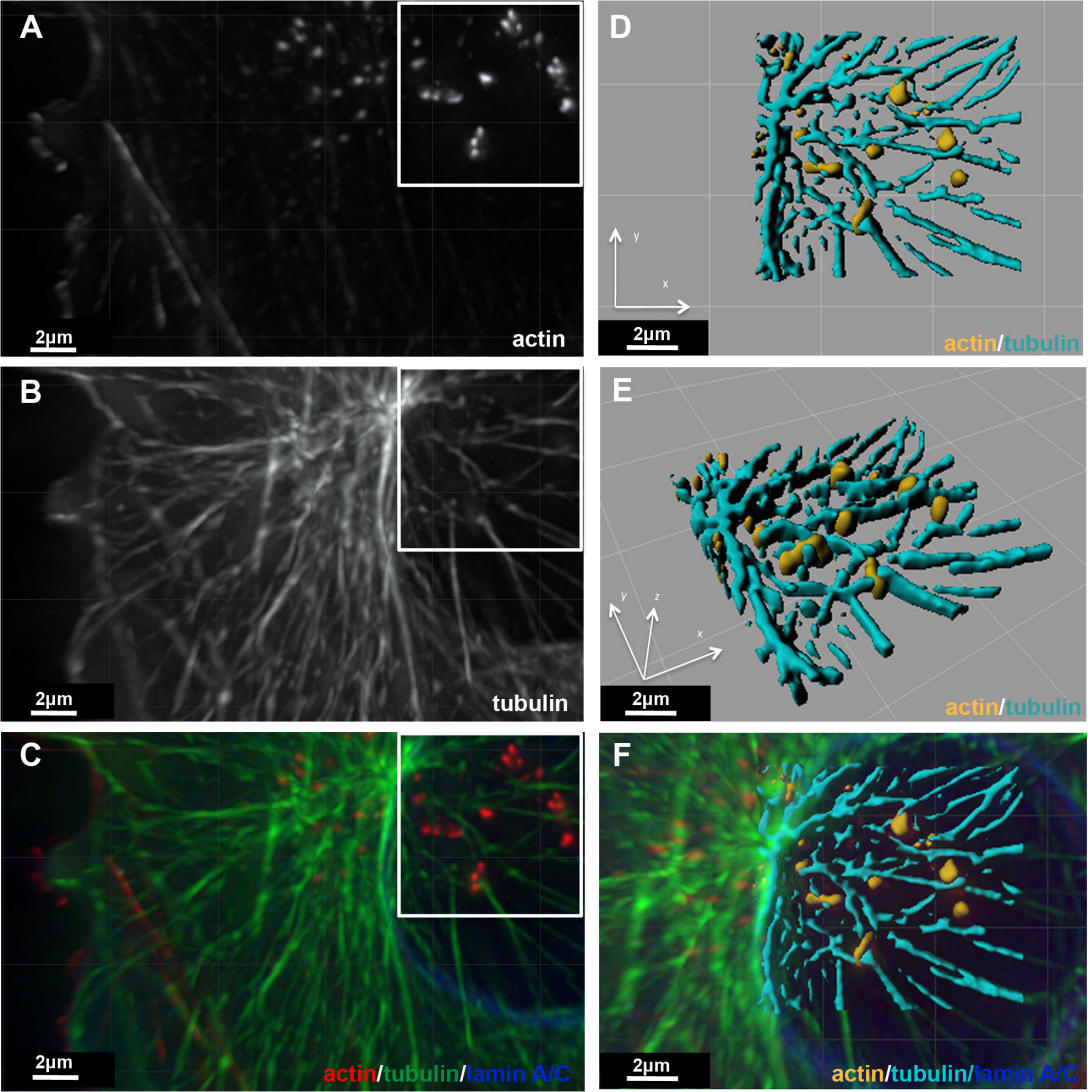
The spatial relationships between microtubules and the core actin bundle of invadopodia. A375 cells, cultured on gelatin and triple labeled for actin (A and C (red)), α-tubulin (B and C (green)) and lamin A/C (blue in Panel C). Z-stacks (0.2 μm apart) of the three colors were acquired, deconvoluted, and 3D reconstruction images were generated (C). Top-down and tilted views of the 3D reconstruction and rendering are shown in (D) and (E), respectively. In (F), the 3D reconstruction image (as in D) is superimposed on the triple-labeled image.

To visualize invadopodia, A375 cells co-transfected with both LifeAct-GFP as an invadopodia marker, and mCherry-vinculin as an adhesion ring marker, were cultured on gelatin-coated gridded glass surfaces. Cells displaying invadopodia and adhesion rings were then identified (Fig. 2A, left image; numbers 1–3) using light microscopy at high magnification (x100/1.3 oil objective), and their coordinates relative to the grid were recorded. The samples were then fixed *in situ*, and embedded in Epon for FIB-SEM imaging (for details, see Materials and Methods). The precise location of the cell in the Epon block was confirmed by the grid imprint on the block. A stack of images with a total thickness of 10 μm was then acquired, using the “slice and view” mode of the FIB-SEM system, with a slice thickness of 10 nm (Supplementary Movie 2). Single, cross-sectioned slices of the FIB-SEM stack (Fig. 2A, right panel; numbers 1 and 2), together with a 3D reconstruction of the whole stack (Supplementary Movie 2), showed that invadopodia cores are cylindrical protrusions of the ventral cell membrane, that are devoid of cytoplasmic organelles. This exclusion of ribosomes and membrane vesicles, present in the surrounding cytoplasm, is attributable to the tight packing of actin fibers in the invadopod core^22^. Using a “top-down view”, of the cell-substrate interface, based on the FIB-SEM reconstruction (Fig. 2B), we visualized the precise location of the vinculin rings at the periphery of the ventral aspect of the invadopod columns (Fig. 2B′ and 2B″). To clearly illustrate the ring localization, and its position relative to the actin core, a magnification of invadopod #3 (as in “B”, shown here in inverted contrast), was super-imposed on a schematic representation of vinculin (red ring) and F-actin (green) immunofluorescence (Fig. 2B′″). Correlated microscopy examination of the cytoplasm in the immediate area surrounding the invadopodia core bundle revealed Golgi and ER membranes, as well as small vesicles (Supplementary Fig. 3) and microtubules (Fig. 3) at the “apical region” of invadopodia, in agreement with previous reports^9^,^10^.

To further study the spatial relationships of invadopodia and the microtubule system, A375 cells were cultured on gelatin-coated dishes, and the 3D organization of their actin filaments and microtubules was reconstructed, based on deconvolution fluorescence microscopy. Examination of the spatial distribution of the two systems indicated that microtubules (Fig. 3B) surround the actin bundles (Fig. 3A), but are consistently excluded from their cores (Fig. 3C). Further rendering and 3D reconstruction also confirmed that spatially, the actin and the microtubule systems are mutually exclusive (Fig. 3D, 3E, 3F; Supplementary Movie 3).

### Detection of a physical interaction between invadopodia and the juxtaposed nucleus

**Figure 4:**
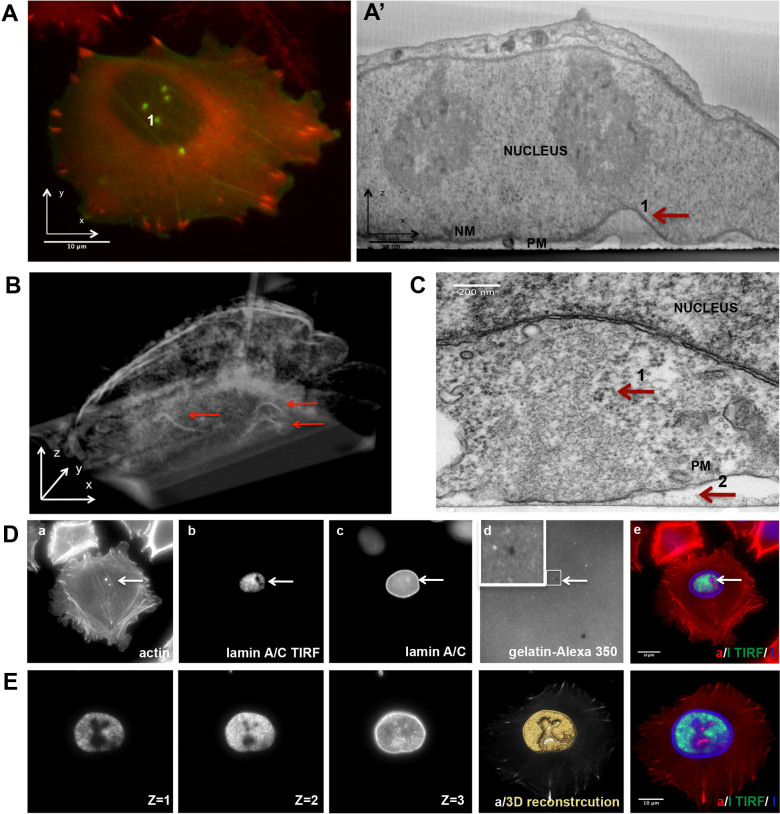
A nuclear indentation detected at the interface between the invadopodia core and the juxtaposed nucleus. (A) A375 cells were transfected with LifeAct-GFP (green) and mCherry-vinculin (red) and plated on gelatin-coated coverslips for 2 h. Fluorescence microscopy of the double-labeled cell is shown in the left panel. The invadopod marked as #1 was further examined by FIB-SEM (A′). A FIB-SEM image revealed an indentation in the nucleus, coinciding with the interface between the dorsal tip of the invadopod core actin bundle and the nuclear membrane (red arrow). (B) 3D reconstruction of the FIB-SEM view of the whole cell presented in (A). Red arrows mark three nuclear indentations. (C) TEM image of an invadopod (side view), showing the core actin bundle (dense area, devoid of organelles) (Arrow 1), the nuclear indentation, and the invasion into the gelatin layer (Arrow 2). (D) A375 cells, cultured for 2 h on a gelatin-Alexa 350-coated glass- bottomed dish. The images (from left to right): (a) actin, marker for invadopodia core. Note the invadopodia, indicated by the arrow; (b) Lamin A/C imaged by TIRF microscopy. The dark region indicated by the arrow corresponds to the invadopodia-associated indentation in the nucleus; (c) Lamin A/C imaged by epi-fluorescence in a higher focal plan, showing an intact nuclear lamina; (d) Fluorescently-tagged gelatin. The dark spot indicated by the arrow corresponds to the area degraded by the invadopod; (e) Overlay of actin (red), Lamin A/C TIRF (green) and Lamin A/C epi-fluorescence (blue). (E) TIRF imaging of the nuclear indentation, using variable TIRF angles and producing evanescence layers of increasing thickness enabling the imaging of the entire indentation. From left to right, three TIRF angles (Z = 1–3) show different focal planes of the indentation. Top-down view of three-dimensional reconstruction of the indentation (yellow), invadopodia-associated F-actin bundle, inside the indentations, is shown in white. The reconstruction is for illustration only and was performed by setting an artificial Z pixel size (in the order of the X-Y pixel size). Triple overlay of actin (red), Lamin A/C TIRF (green) and Lamin A/C epi-fluorescence (blue).

Examination of A375 melanoma cells, as well as other cultured cancer cells, shows that invadopodia tend to form in the vicinity of the nucleus^8^,^9^,^10^; yet the reason for this spatial distribution is unclear. Mapping of the spatial distribution of invadopodia in cultured A375 cells indicated that over 80% of invadopodia are located under or in the immediate vicinity of the nucleus (Supplementary Fig. 4). Such distribution suggests that there might be some physical interaction between invadopodia and the nucleus. Indeed, our examination of the reconstructed invadopodia-nucleus interface, based on the correlative light-FIB-SEM technique, indicated that the apical tips of most invadopodia co-localize, with conspicuous (400–500 nm deep) indentations into the nucleus (Fig. 4A, 4A′ number 1; Supplementary Movie 4). Three-dimensional image reconstructions indicated that such indentations (Fig. 4B, red arrows; Supplementary Movie 5) were commonly associated with invadopodia.

To further characterize the interface between the core actin bundle, the nucleus, and the plasma membrane, we examined these areas by Transmission electron microscopy (TEM), which offers higher resolution than that currently attainable by FIB-SEM. The resulting images demonstrated that the apical tip of the actin core bundle indeed touches the nuclear envelope in the indented area (Fig. 4C, Arrow 1). At the basal end of invadopodia, the plasma membrane clearly invades the thin (100 nm) gelatin layer to which it adheres (Fig. 4C, Arrow 2). Tightly packed actin fibers fill the entire space between the ventral cell membrane and the nucleus, excluding other cytoplasmic structures from that region.

Nevertheless, to monitor the invadopod-nucleus interface in a large number of cells, either fixed or live, we explored possibilities for visualizing this region by light microscopy, given the fact that the indentations are small, and challenging to image with epi-fluorescence microscopy. We found that we could visualize the indentations by TIRF microscopy by changing the TIRF angle, thereby modifying the thickness of the evanescence layer. For that purpose, we either fixed and fluorescently immunolabeled the cells for the nuclear lamina markers lamin A/C, or expressed GFP-tagged lamin B1 for live-cell imaging. Examination of lamin labeling in the vicinity of invadopodia indicated that in over 85% of the cases (n = 100), lamin labeling was absent from invadopodia within the TIRF plane, due to the nuclear indentation (Fig. 4D, Image b), while in regular epi-fluorescence microscopy, the nuclear lamina was homogeneously labeled (Fig. 4D, Image c). Changing the TIRF angle, thereby changing the thickness of the evanescence layer, enabled the visualization of the entire indentation and the reconstruction of an illustrative 3D animation (Fig. 4E; Z = 1–3; Supplementary Movie 6). Moreover, such nuclear indentations were also seen on collagen surface and in MDA-231 breast cancer cells (Supplementary Fig. 5a, 5b), suggesting that the nuclear indentation is a common process, displayed by the vast majority of invadopodia in different cell types.

**Figure 5:**
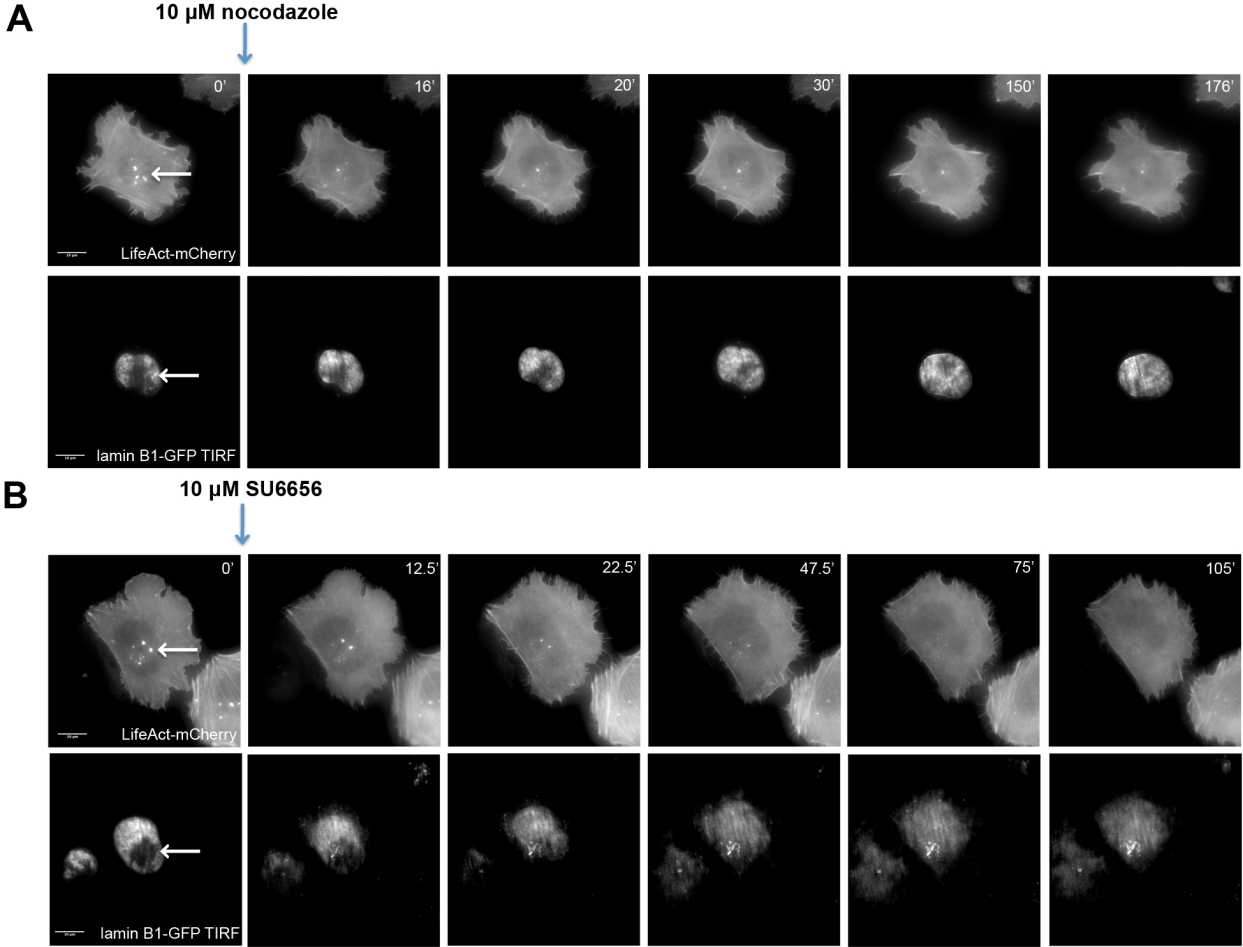
Coordinated modulation of invadopodia and nuclear indentation by microtubules and Src inhibitors. A375 cells expressing LifeAct-Cherry and Lamin β1-GFP cultured on gelatin-coated coverslips for 1 h, then imaged every 2 min for 3 h, using regular epi-fluorescence microcopy for actin, and TIRF microscopy of lamin β1-GFP, for visualization of the nuclear indentation. (A) Addition of nocodazole caused invadopodia disassembly, and a gradual disappearance of the nuclear indentation. Invadopodia and nuclear indentations are marked by white arrows. (B) A similar experiment, in which the Src inhibitor SU6656 was added to induce invadopodia disassembly, resulting in a concomitant loss of invadopodia and of nuclear indentations.

To further study the relationships between invadopodia formation and the nuclear indentation, A375 cells expressing mCherry-actin and GFP-lamin B1 were monitored by TIRF time-lapse microscopy for invadopodia structures and their associated nuclear indentations, and were treated with 10 μM nocodazole (microtubule inhibitor) or 10 μM SU6656 (pp60^src^ inhibitor), (Fig. 5A, 5B). Previous studies demonstrated that the two treatments induce invadopodia disassembly and block matrix degradation^18^,^19^,^20^. Indeed, both treatments induced invadopodia destruction within minutes, followed by loss of the nuclear indentations, and eventual nuclear flattening (Fig. 5A, 5B; Supplementary Movie 7), suggesting a direct interaction and mechanical coupling between the actin cores of invadopodia and the nucleus. Interestingly, upon washout of nocodazole, new invadopodia were reformed in the juxtanuclear area, not always coinciding with the “old”, disassembled ones. These invadopodia formed within 4 minutes of nocodazole washout; concomitantly with their formation, new nuclear indentions, juxtaposed with the invadopodia, became apparent (Supplementary Fig. 6 and Supplementary Movie 8). Another interesting phenomenon shown in Supplementary Movie 8 is the disassembly of invadopodia following translocation of the juxtaposed nucleus, providing further support to the notion that the interaction of invadopodia with the nucleus increases their stability.

### Calculation of the force applied to the nucleus by invadopodia

The results described above demonstrate that invadopodia can push against the nucleus, and deform it. This raises the possibility that this mechanical coupling might enhance the capacity of invadopodia to penetrate into the underlying matrix. To test this hypothesis, we calculated the expected contribution of the nuclear indentation induced by the elongating actin bundle, to the mechanical forces applied by the invadopodia to the ECM.

The underlying physical principle used for this calculation is that when a hard solid is pressed against a softer one, the latter will deform according to its elastic properties, the contact geometry, and the applied force. In this manner, when two of the relevant parameters are controlled (typically, the applied force and the contact geometry), the third parameter (in this case, the elastic properties of the softer material) can be inferred from the resulting indentation.

Our hypothesis was that the relatively rigid actin bundle of the invadopodia indents the considerably softer nucleus (the rigidity of actin filaments is ~2 GPa^29^, vs. ~5 kPa for the nucleus^30^,^31^,^32^,^33^). In order to calculate the degree of force that individual invadopodia apply on the nucleus, we set out to analyze the deformation patterns of the nuclear envelope. These should reflect, in a well-defined manner, the invadopodia-nucleus contact geometry, which could not be observed directly. Therefore, as a preliminary step, it was necessary to determine which contact geometry could account for the entire indentation profile of the observed nuclear envelope deformations (including depressed regions which are outside the contact area itself).

**Figure 6:**
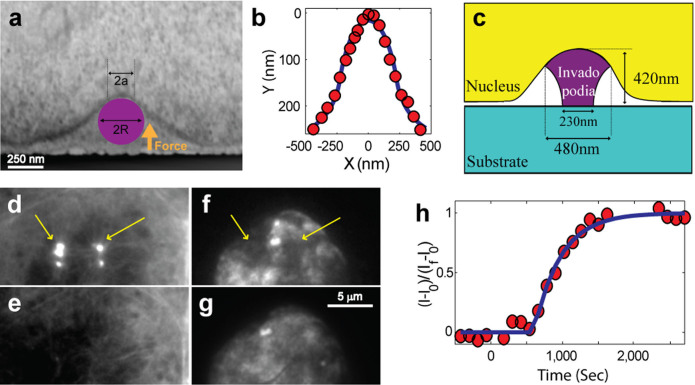
Calculation of the force applied on the nucleus by a single invadopod actin core. (a) One slice of a correlative fluorescence-FIB-SEM stack of A375 cells, show the nuclear indentation. A spherical indenter of radius R, pushing onto the cell nucleus with a force F, can account for the observed nuclear deformation. The diameter of the contact region of the invadopod with the nucleus is defined as 2a. (b) Measurement of the nuclear deformation pattern in (a): The Y axis represents the height, whereas the X axis corresponds to the distance along the cell-substrate “serum line”. Solid red circles correspond to the nuclear depression pattern [dark grey curve in (a)], while the blue line indicates the best fit for the analytical solution of a spherical indenter (n = 3). (c) The resulting invadopodia shape has a circular base, which widens almost two-fold at the spherical cap (n = 3). (d–g) Snapshots of the time-lapse microscopy presented in Figure 5. (d) Actin structures of invadopodia (yellow arrows). (f) TIRF microscopy of Lamin-B1, showing that the nucleus is deformed (yellow arrows). (e) When treating cells with 10 μM nocodazole, the invadopodia actin cores disassemble within 2 minutes. This is accompanied, as expected for a mechanical interaction, by a straightening of the cell nucleus (g), albeit at considerably longer time-scales (~20 minutes). Remarkably, the flattening-out dynamics of the nuclear envelope (characterized by its fluorescence intensity - I), follows a simple exponential relaxation (h), with a viscoelastic timescale of t = 340 seconds (n = 4). Solid red circles mark normalized intensity measurements, while a blue line is the best fit to the equation 
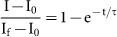
, with I_f_ and I_0_ the final and initial intensities, respectively. Cells were treated with nocodazole at timepoint t = 0.

To characterize the invadopod-nucleus contact geometry, we extracted the peak cross-section profiles of three nuclear depressions (each associated with the actin core bundle of a single invadopod), derived from correlative light-FIB-SEM images. Testing for different indenter geometries and assuming no additional sources of force, we found that the analytical solution for a spherical indenter^34^ (Fig. 6A) provides an excellent fit for the measured deformation profiles (Fig. 6B). Moreover, the invadopod shape, which emerges from this analysis and the FIB-SEM images, appears quite uniform in its dimensions. Extending from a circular base with an average diameter of ~230 nm (min-max range: 220–250 nm) in contact with the plasma membrane, the individual invadopod reaches a height of ~420 nm (350–500 nm), and expands as a spherical cap of an ~480 nm (460–520 nm) cross-section in contact with the nuclear envelope (Fig. 6C).

With the exact deformation profile of the nucleus and its contact geometry to the invadopodia both at hand, we proceeded to calculate the magnitude of the applied indentation force^34^. Interestingly, it would be the same degree of force applied by the invadopodia to the underlying matrix (based on force balance in a serial system, where it is assumed there is no other mechanical interaction of the invadopodia besides the nucleus at their apex, and the plasma membrane at their base). Thus, using typical values of cell nucleus elastic parameters (Young's modulus of ~5 kPa; Poisson's ratio of ~0.5^30^,^31^,^32^,^33^,^35^) we found that each invadopod presses down on the substrate (away from the cell center) with a force of ~1 nN, which translates to a stress of ~20 nN/μm^2^ (~20 kPa). As the contact area of the invadopodia with the nucleus is 4–5 fold greater than that with the plasma membrane, the pressure exerted on the nucleus by the same (indentation) force is greatly reduced, being distributed over a larger contact region (see Fig. 6C for invadopod contact dimensions with the nucleus and the plasma membrane).

Further analysis of the timescales of nuclear flattening, followed by invadopodia disassembly after nocodazole or src inhibitor treatment (Fig. 5A, 5B; Supplementary Movie 7) shows that while the actin core of invadopodia effectively vanishes within 2 minutes of such treatment (Fig. 6D), the indented nuclear envelope responded to the stress relief on a much slower timescale. It apparently began to straighten out only ~15 minutes later, gradually returning to its undeformed state (Fig. 6F–6H). This finding indicates a highly viscoelastic response, consistent with previous measurements of cell nuclei elastic properties^36^,^37^.

### LINC complex inhibition reinforces invadopodia adhesion to the matrix

Physical interactions of the nucleus with the cytoskeleton, and transmission of mechanical signals from the plasma membrane toward the nucleus, are mediated via the LINC (linkers of the nucleoskeleton to the cytoskeleton) complex, which is associated with the nuclear envelope^12^,^28^,^29^,^30^. To explore the possibility that the LINC complex is involved in the interactions of invadopodia with the nucleus, we mapped the distribution of the LINC complex components sun1, sun2 and nesprin-2, relative to invadopodia, and tested the effects of siRNA-induced downregulation of LINC proteins on invadopodia formation, function and structure.

**Figure 7:**
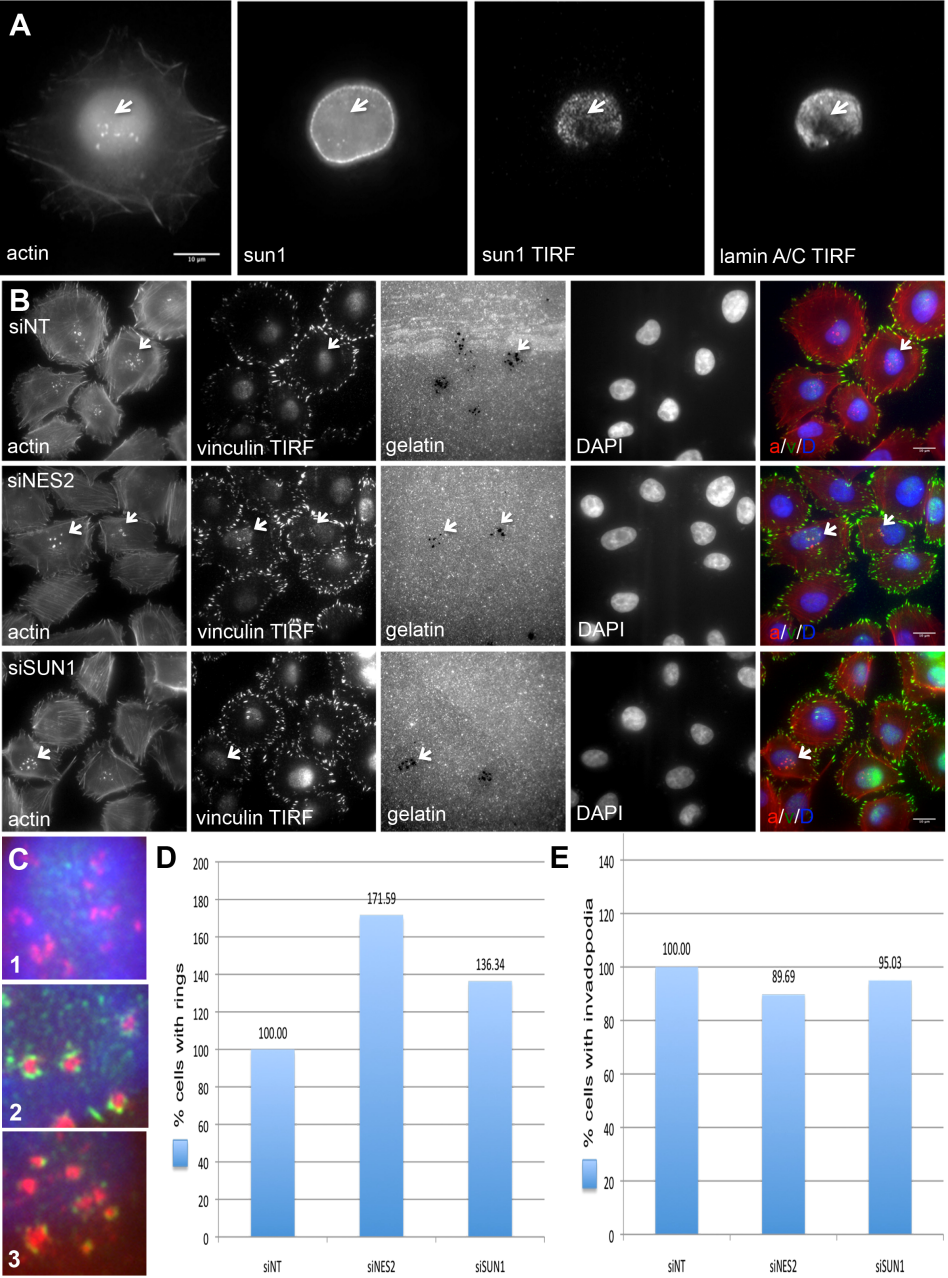
LINC complex inhibition increases invadopodia adhesion to the matrix. (a) A375 cells were cultured on gelatin-coated glass-bottomed dishes for 3 h, than fixed and stained for actin, lamin A/C and SUN1. From left to right: Actin staining (invadopodia structures denoted by white arrows), SUN1 staining in the nuclear lamina, and SUN1 TIRF and lamin A/C TIRF microscopy, showing the nuclear indentations. (b) A375 cells were transfected with non-targeting siRNA (siNT), siNESPRIN-2, or siSUN1, and cultured for 3 h on fluorescently labeled gelatin-coated glass-bottomed dishes. Cells were stained for actin, vinculin, and DAPI. White arrows denote invadopodia structures, their respective adhesion vinculin ring, and matrix degradation. On the right, an overlay of actin (a, red), vinculin (v, green), and DAPI (D, blue). (c) Magnification of the triple overlay (as in b) 1 = siNT, 2 = siNESPRIN-2, 3 = siSUN1. (d) Quantification of the cells that form adhesion rings in siNT (n = 141), siNESPRIN-2 (n = 131) and siSUN1 (n = 155) transfected cells. The results are representative of findings in three repeating experiments. (e) Quantification of the cells that form invadopodia in siNT, siNESPRIN-2, and siSUN1 transfected cells. The results are representative of findings in three repeating experiments.

As shown in Fig. 7A, the proteins tested displayed a broad distribution throughout the nuclear membrane (only SUN1 is presented in the Figure), and were clearly visible both within and outside the invadopodia-induced indentations.

To search for a possible function of the LINC complex in invadopodia structures, we knocked down either SUN1 or nesprin-2 by siRNA (Supplementary Fig. 7), and measured invadopodia formation, function and adhesion to the matrix (Fig. 7B–7E). Interestingly, the KD of these LINC complex components did not significantly affect invadopodia formation (Fig. 7E) or degradation of the underlying gelatin matrix (data not shown); yet the prominence of invadopodia associated with vinculin-rich adhesion rings increased by 70% and 35%, following the knockdown of nesprin-2 and SUN1, respectively (Fig. 7D). These results demonstrate that LINC complex inhibition can exert long-range effects on invadopodia, as manifested in the enhancement or stabilization of their matrix adhesion domains.

## Discussion

In recent years, a number of studies have associated invadopodia with ECM invasion and dissemination of metastases by malignant cells^38^,^39^,^40^. The classical “grip, degrade and push” model^27^,^41^ suggests that the protrusive activity of invadopodia depends on a coordinated process that includes local adhesion to the matrix, proteolytic degradation of the underlying ECM by metalloproteinases, and membrane pushing, driven by Arp2/3^12^,^42^,^43^,^44^ or formins^28^ actin polymerization. Both actin nucleation mechanisms are known to drive protrusive processes, such as extension of lamellipodia or filopodia^45^,^46^,^47^,^48^; yet the mechanical challenge faced by invadopodia-driven penetration into the ECM is considerably more demanding, given the expected resistance of the matrix to external mechanical perturbations^49^,^50^.

**Figure 8:**
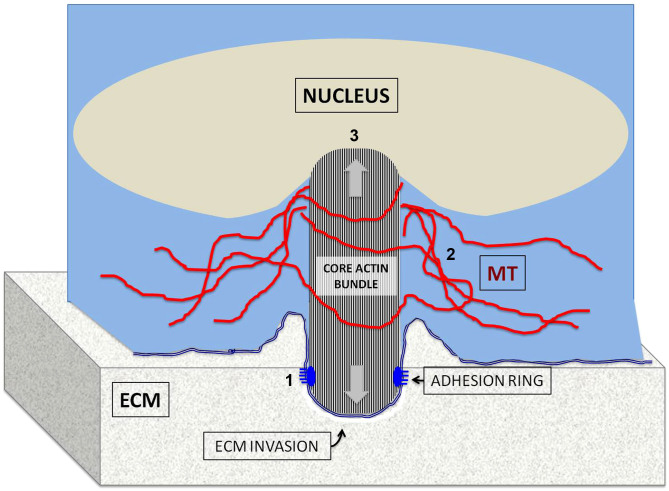
Schematic representation of the different structural domains of invadopodia. In this scheme, a single invadopod is presented, penetrating into the extracellular matrix (ECM) and anchored to it via an adhesion ring (1), located at the peripheral edge of the protrusion, interfacing the ECM. As indicated in Supplementary Fig. 1, the adhesion ring is predominantly found at early stages of invadopod formation. The protrusion is filled with a core actin bundle that polymerizes at the protrusion tip, pushing it towards the ECM (arrow pointing downwards). The “dorsal aspect” of the core bundle elongates towards the nucleus (arrow pointing upwards), and indents it (3). It is proposed that actin polymerization, confined by the protrusion tip and the nucleus, generates the mechanical force needed for penetration into the ECM. Along its entire length, the actin core is surrounded, and possibly stabilized, by a web of microtubules (2).

To explore the mechanical elements used by invadopodia in order to overcome the physical challenges of invading a matrix, typically as rigid as the cell itself^37^, we examined the interactions between the invadopodia's main force generation device; namely, the actin core, with diverse cellular compartments, using correlative imaging approaches that combined fluorescence and TIRF microscopy with high-resolution EM and FIB-SEM technologies. Specifically, we focused on interactions mediated via three distinct structural domains of invadopodia: their basal region, where they interface with the ECM; their central region, which extends through a web of microtubules; and their dorsal region, which, as we show here, tends to terminate at an indentation in the nuclear envelope. These features of invadopodia are presented schematically in Figure 8, and their functional significance is discussed below.

The role of the adhesive interactions of invadopodia with the underlying ECM is a rather enigmatic issue. Conceptually, invadopodia are matrix-degrading organelles, and thus would be expected to destabilize or even directly destroy their own cell-matrix adhesions. Indeed, previous studies suggested that invadopodia do not form stable adhesive structures^5^,^6^. Nevertheless, this claim was challenged lately by several studies demonstrating that invadopodia could display a prominent adhesion domain containing classical adhesome components, among them vinculin, paxillin and Hic-5^23^,^24^. In this study, we confirm the presence of an adhesion ring around the ventral aspect of the actin bundle, and show that unlike related adhesions, such as podosomes^3^, the majority of invadopodia formed prominent adhesion rings within 1 hour after plating, together with the core formation; yet most of the rings were lost when incubated for longer periods (typically, 6 hours; see Fig. 1A, Supplementary Fig. 1), in agreement with Branch *et al*^23^. Whether this loss of adhesion is caused by local proteolytic degradation of the matrix, or by other changes that take place upon invadopod maturation, is unclear. We further show here that this adhesion ring is confined to the interface of the actin bundle and the plasma membrane, while the bundle itself elongates towards the apical direction (Fig. 1B, 2B′″, shown schematically in Fig. 8; number 1).

The transient appearance of an adhesion ring while local matrix degradation is still ongoing is intriguing. Is the mechanical support not essential for the activity of “mature invadopodia”, or is it provided by other cellular mechanisms?

Possible clues for additional mechanical supports became apparent upon systematic examination of the cytoplasmic organization around the actin core of invadopodia, by a combination of electron and light microscopy techniques. As shown here, the central regions of single or clustered invadopods are commonly flanked by a dense web of microtubules (shown in Fig. 3 and schematically, in Fig. 8, number 2). Interestingly, despite their prominence around invadopodia, microtubules are excluded from the actin-rich core (Fig. 3). Indeed, microtubule disruption induces rapid disassembly of invadopodia and inhibits matrix degradation in melanoma cells^20^,^21^,^22^. Microtubules were also suggested be important in the polarized transport of MMPs to invadopodia^20^. Whether microtubules are needed for the direct mechanical support of invadopodia or whether they have a role in the transport of MMPs or integrin, remains unclear.

The most intriguing and striking observation reported in this study is the apparent physical interaction between the “dorsal tip” of the invadopod core bundle, and the nucleus, leading to the development of deep indentations (~500 nm) in the nuclear membrane (presented schematically in Fig. 8, number 3). These indentations were initially identified by correlative light-FIB-SEM microscopy (Fig. 4A, 4A′), and then confirmed by TEM imaging (Fig. 4C) and TIRF microscopy in fixed and live cells (Fig. 4D). The monitoring of nuclear indentations by TIRF microscopy enabled the examination of a large number of invadopodia, indicating that nuclear indentations exist in over 85% of invadopodia in A375 melanoma cells. Such indentations were also seen in MDA-231 breast cancer cells (Supplementary Fig. 5a) and on other matrix surfaces such as collagen (Supplementary Fig. 5b). Moreover, disassembly of invadopodia induced by microtubules and src inhibitors lead to nuclear flattening (Fig. 5A, 5B). Further live-cell experiments in which the inhibitor (nocodazole) was washed out, and the *de novo* formation of both new invadopodia and nuclear indentation, was monitored by TIRF microscopy in real time, indicated that the two developed essentially simultaneously (Supplementary Fig. 6, and Supplementary Movie 8). Furthermore, occasional nuclear translocation away from invadopodia leads to destabilization of the underlying invadopodia and, eventually, to their disassembly. All in all, these results support the view that the elongating actin core bundle pushes against the nucleus, mechanically interacts with it and indents it and that forces exerted by the deformed nucleus stabilize invadopodia, and block their disassembly.

Using the nuclear depression patterns extracted from the correlative light-FIB-SEM microscopy images, and reported values of the cell nuclei's elastic properties^30^,^31^,^32^,^33^, we estimated the force that the a single actin core of invadopodia applies to the nucleus to be on the order of ~1 nN (Fig. 6). Based on force balance, this would be the same degree of force applied by invadopodia on the underlying matrix. This force translates to a stress of ~20 nN/μm^2^ (~20 kPa), based on the contact area of invadopodia with the matrix (a circular base with an average diameter of ~230 nm; Fig. 6C). These stress values are on the order of, or even higher than, the rigidity values reported for mesenchymal connective tissues^51^, and thus, can be relevant to the role of invadopodia in their penetration into the matrix.

In this study, we further explored the involvement of the LINC complex in interactions between invadopodia and the nucleus (Fig. 7A)^12^,^28^,^29^,^30^. Interestingly, knockdown of LINC complex components did not significantly affect invadopodia formation (Fig. 7E) or matrix degradation (data not shown); yet the prominence of the invadopodia-associated adhesion rings increased by 70% and 35%, following knockdown of nesprin-2 and SUN1, respectively (Fig. 7B, 7C, 7D). These results demonstrate that LINC complex inhibition can exert long-range effects on invadopodia, as manifested in the enhancement or stabilization of their matrix adhesion domains, and suggest direct molecular interactions between the actin bundles of invadopodia, and the nuclear membrane. The fact that invadopodia formation and matrix degradation were not altered in LINC complex KD cells, while matrix-mediated adhesions increases, is consistent with the possibility that invadopodia have alternative and cross-coordinated mechanisms for mechanical support and stabilization: once one becomes ineffective, the other compensates for its lack of activity.

We further demonstrate that invadopodia structures have clear basal and apical regions. The basal region of the adhesion anchors the protrusion to the ECM in a transient fashion. This anchoring is supported, or replaced, by the anchoring of the bundle to the apical region of the nuclear membrane.

The nuclear indentations caused by the elongating actin bundle of invadopodia bear an obvious relevance to the “classical” function of invadopodia; namely, ECM invasion, but with a novel underlying physical mechanism. It is known that the actin cytoskeleton generates a mechanical coupling between forces that are applied to the cell membrane and the nucleus^52^,^53^,^54^,^55^. It is conceivable that the nucleus, being a rigid, central organelle in the cell^56^, together with invadopodia localization under the nucleus, can contribute to the directional invasion generated by the polymerizing actin protrusion, essentially coupling the protrusive edge of the cell to the cell body. Recently, Petrie *et al.* described a similar scenario, in which the nucleus can be pushed by actomyosin contractility in order to generate pressure on the leading edge of 3D migrating cells, thus promoting lobopodia formation^57^. Nuclear indentation and pushing by microtubules was also shown recently to be important in dorsal-ventral axis formation in *Drosophila*^58^.

Moreover, it is worth noting that beyond its mechanical impact on the matrix, deformation of the nucleus can also affect nuclear functionality^52^, including processes of transport into and out of the nucleus, as well as transcriptional activity^55^ and cell division^26^.

Evidently, in our experiments, the underlying matrix (gelatin-coated glass) is considerably stiffer than the nucleus (~70 GPa for glass, vs. ~5 kPa for the nucleus), allowing only limited compression of the gelatin coat (Fig. 4C). Yet *in vivo*, typical matrix rigidities fall within the range of 1–10 kPa (notable exceptions being bone, which is much stiffer, or the brain, which is much softer)^37^, similar to measurements of nuclear stiffness^30^,^31^,^32^,^33^. This suggests that purely mechanical invadopodia penetration into the underlying ECM should be comparable to its indentation of the nucleus (i.e., ~0.5 μm in our system). Conversely, when the ECM is much softer than the nucleus, or highly porous^22^, the cell membrane and not the matrix provides the only elastic force resistant to invadopodia growth. As a consequence, invadopodia protrusion may be significantly deeper (by analogy to filopodia in 2D, which, like invadopodia, are spike-like actin protrusions, we can estimate the maximum invadopodia protrusion into a soft ECM to be ~10 μm long).

Taken together, the results presented here provide novel insights into the mechanical penetration of invadopodia structures into the matrix, and suggest a mechanism for their directed matrix invasion.

## Methods

### Antibodies, plasmids and reagents

The Src inhibitor SU6656 was purchased from Sigma (St. Louis, MO, USA) and was used at concentrations of 10 μM. The microtubule inhibitor nocodazole (Sigma) was used at concentrations of 10 μM.

The antibodies used in this study included: Mouse monoclonal anti-vinculin antibodies, clone hVin-1 and anti α-tubulin, DM1A (Sigma); mouse monoclonal anti-paxillin and anti-zyxin antibodies (BD Transduction Laboratories, San Jose, CA, USA); rabbit polyclonal anti-TKS5 antibodies, and mouse monoclonal antibodies to integrin β1and to ILK (Santa Cruz Biotechnology, Santa Cruz, CA, USA); rabbit polyclonal anti-integrin β3 (Abcam, Cambridge, UK); goat anti-mouse IgG conjugated to Alexa Fluor 488 (Invitrogen, Carlsbad, CA, USA); goat anti-mouse IgG conjugated to Cy5, and goat anti-rabbit IgG conjugated to cy3 (Jackson ImmunoResearch Laboratories, Inc., West Grove, PA, USA). Mouse monoclonal anti-lamin A/C, rabbit polyclonal anti-lamin C, and lamin B1-GFP were kindly provided to us by Prof. Harald Herreman, German Cancer Research Center (DKFZ), Heidelberg, Germany. Monoclonal nesprin-2, and polyclonal SUN1 and SUN2 antibodies were kindly provided to us by Dr. Sue Shackleton, University of Leicester, Leicester, UK.

F-Actin was fluorescently labeled with TRITC-phalloidin (Sigma).

GFP-LifeAct, Cherry-LifeAct and mCherry-vinculin were obtained from the Davidson plasmid library (Davidson College, Davidson, NC, USA).

Cells were transfected using Lipofectamine2000 (Invitrogen) according to the manufacturer's instructions, or by using a Neon® Electroporator (Life Technologies, Carlsbad, CA, USA) at 1050 mA, using 2 pulses, 30 seconds each, for higher transfection efficiency. Cells were cultured for 24 h in complete medium, and then replated onto glass-bottomed plates (MatTek Corp., Ashland, MA, USA), coated with fluorescent gelatin or gelatin gel (see below), and cultured for varying lengths of time (mostly 1–2 h), then either fixed, or subjected to live-cell video microscopy. siRNA transfection of siGENOME SMART pool (Dharmacon, CO, USA) was performed using Lipofectamine 2000 (Invitrogen) in a 20 μM concentration. In every transfection, siNON-TRAGETING was used as control, and siTOX as the transfection efficiency reporter. Cells were incubated for 48 h before replating for an experiment.

### Cell cultures

A375 metastatic melanoma cells and MDA-231 metastatic breast carcinoma cells were obtained from the American Type Culture Collection (ATCC) (Manassas, VA, USA) and cultured in DMEM supplemented with10% FCS (Gibco, Grand Island, NY, USA), 2 mM glutamine, and 100 U/mL penicillin-streptomycin. Cultures were maintained in a humidified atmosphere of 5% CO_2_ in air, at 37°C. For imaging, cells were cultured for 2 h on 13 mm matrix-coated coverslips or 35 mm glass-bottomed dishes (MatTek Corporation, catalogue# P35G-0-14-C).

### Quantitative real-time PCR (QRT–PCR)

Total RNA was isolated using an RNeasy mini-kit (Qiagen, Valencia, CA, USA), according to the manufacturer's protocol. A 2 μg aliquot of total RNA was reverse transcribed, using a high-capacity cDNA reverse transcription kit (Applied Biosystems, Carlsbad, CA, USA). Quantitative real-time PCR (QRT–PCR) was performed with a OneStep instrument (Applied Biosystems), using Fast SYBR® Green Master Mix (Applied Biosystems). Gene values were normalized to a GAPDH housekeeping gene. The following primers were used: nesprin-2 F 5′ GTGGTCTCTGTCAACGTGAGC 3′, R 5′ GAGCGACTGTCGTAAGCCC 3′. Sun1 F 5′ ACGTATGCGCTCAGTTCCAG 3′ R 5′ GCA AACTACGGCGGGACATC 3′. GAPDH F 5′ TGGCACCGTCAAGGCTGAGA 3′, R 5′ TTGGCTCCCCCCTGCAAATG 3′.

### Gelatin coating

Gelatin gel: coverslips (13 mm diameter) or glass-bottomed 35 mm dishes (MatTek Corp.; catalogue # P35G-0-14-C), were coated with 50 μg/ml poly-L-lysine solution (Sigma, catalogue # P-7405) in Dulbecco's PBS, and incubated for 20 min at room temperature. The coverslips or dishes were then gently washed 3 times with PBS. Porcine skin gelatin, 0.2 mg (Sigma, catalogue #G2500) was dissolved at 37°C in 100 ml ddH_2_O. Gelatin was cross-linked with 1-ethyl-3-(3-dimethylaminopropyl) carbodiimide (EDC, Sigma, catalogue #03450) and N-hydroxysuccinimide (NHS, Sigma, catalogue #130672), each prepared as 10% solutions in ddH_2_O. Glass-bottomed 35 mm dishes were coated with 100 μl of gelatin and cross-linker mixture, and 13 mm coverslips were inverted onto a drop of 40 μl of the mixture. The ratios of gelatin to cross linkers were 82.5:12.5:5 (gelatin:NHS:EDC).

Surfaces were incubated for 1 h, and sterilized by 30 min of UV radiation.

### Collagen coating

Collagen gel: rat tail type I collagen (Roche, Mannheim, Germany) was dissolved in acetic acid to obtain a 3 mg/ml solution. Collagen was cross-linked to coverslips (13 mm diameter) or glass-bottomed 35 mm dishes (MatTek catalogue # P35G-0-14-C), as described above for gelatin gel.

### Gelatin degradation assay

Glass coverslips coated with fluorescent gelatin were prepared, as previously described^59^,^60^. Acid-washed coverslips were first coated with 50 mg/ml poly-L-lysine for 20 min at room temperature, then washed and treated with 0.5% glutaraldehyde for 15 min, and washed with PBS. Gelatin matrix was prepared by mixing 0.2% porcine skin non-labeled gelatin (Sigma, catalogue #G2500) with Alexa-488 (1:8 ratio), Alexa-350 (1:3 ratio) or Alexa 660 (1:3 ratio)-labeled gelatin (protein labeling kit; Invitrogen). The treated coverslips were inverted onto a 40 μl drop of the gelatin mixture for 10 min, then washed with PBS, and reduced (15 min) with 5 mg/ml of sodium borohydride, followed by extensive washing and UV sterilization. The same protocol was applied to glass-bottomed 35 mm and glass-bottomed multi-well plates. For degradation assays, cells were plated on the gelatin matrix, and cultured for varying lengths of time. Cells were fixed and stained, and degradation area was assessed.

### Immunofluorescence staining, immunofluorescence microscopy, and image analysis

For immunostaining, cells were plated at ~70% confluence on gelatin-coated coverslips or glass-bottomed dishes (see above) for varying time periods. Cells were fixed for 3 min in warm 3% PFA (Merck, Darmstadt, Germany), 0.5% Triton X-100 (Fluka-Chemie AG, Switzerland), followed by 3% PFA alone for an additional 30 min. After fixation, cells were washed 3 times with PBS and incubated with primary antibody for 1 h, washed 3 times in PBS, and incubated for an additional 30 min with the secondary antibody, washed again, and either mounted in Elvanol (Moviol 4–88; Hoechst, Frankfurt, Germany) or left in PBS for TIRF imaging or Z stack acquisition. Images were acquired using the DeltaVision Elite system (Applied Precision Inc., Issaquah, WA, USA), using 100x/1.3 or 60x/1.42 oil objectives (Olympus, Tokyo, Japan). Total internal reflection (TIRF) microscopy was carried out with the DeltaVision system, using 100x/1.49 or 60x/1.42 TIRF oil objectives (Olympus). Image analysis was performed using the UCSF PRIISM environment (http://msg.ucsf.edu/IVE), ImageJ software (rsbweb.nih.gov/ij) and Amira software (FEI) (http://www.vsg3d.com/amira/overview). Z stacks were deconvoluted by DeltaVision software (Applied Precision, Inc.), and analyzed by Imaris software (http://www.bitplane.com/go/products/imaris).

### Time-lapse movies

For time-lapse movies, cells were transfected with the relevant constructs. Cells were cultured overnight, and then replated on 35 mm glass-bottomed dishes coated with one of the gelatin substrates (see above), and cultured for 1–2 h to enable cell spreading. Images were acquired by DeltaVision (RT or Elite) microscopes, using 100x/1.3 or 60x/1.42 oil objectives (Olympus), or by a DeltaVision Elite microscope equipped with total internal reflection (TIRF) optics (Applied Precision, Inc.) using 100x/1.49 TIRF or 60x/1.42 oil objectives (Olympus). The system is equipped with a temperature- and CO_2_-controlled environmental box.

### Electron microscopy

#### Transmission Electron Microscope (TEM)

For TEM imaging, cells were cultured for 2 h on gelatin-coated 35 mm plates (MatTek Corporation, catalogue # P35G-0-14-C). Cells were fixed with warm fixative [3% paraformaldehyde, 2% glutaraldehyde, 5 mM CaCl_2_ in 0.1 M cacodylate buffer (pH 7.4)] for 1 h. Samples were then washed 3× for 5 min each in 0.1 M cacodylate buffer, and post-fixed with 1% OsO_4_ (EMS, Hatfield, PA, USA), 0.5% potassium dichromate, and 0.5% potassium hexacyanoferrate in 0.1 M cacodylate buffer for 1 h. Samples were washed again with 0.1 M cacodylate buffer and ddH_2_O, and stained with 2% aqueous uranyl acetate (EMS) for 1 h. Sample dehydration was performed in increasing concentrations of reagent-grade ethanol (25–95%), followed by 2× 100% ethanol for 10 min each. Dehydration was followed by gradual substitution of the ethanol in the samples with Epon, followed by baking at 60° for 3 days. The coverslip was dissolved by treatment with 30% fluoric acid for 2 h. Epon blocks were cut into small pieces, and re-embedded in the desired orientation. Finally, 10 nm cross-sectioned slices were cut, mounted onto grids, and imaged by an FEI Tecnai™ Spirit T12 transmission electron microscope (FEI Company, Hillsboro, OR, USA).

#### Correlative Fluorescence-FIB-SEM

Cells were cultured for 2 h on gridded glass-bottomed dishes (MatTek Corporation, catalogue #P35G-2-14-C-GRID No. 2) and coated with gelatin gel, as described above. Cells were then fixed with warm fixative (3% paraformaldehyde, 2% glutaraldehyde, 5 mM CaCl_2_ in 0.1 M cacodylate buffer (pH 7.4) for 1 h, and reduced with 5 mg/ml sodium borohydride for 15 min, followed by extensive washing. Cells were imaged by the DeltaVision system, using 20× or 100×/1.3 oil objectives (Olympus). Epon blocks were prepared as described above, and separated from the gridded glass by pre-heating the glass to 62°C for 3.5 min, and fast cooling in liquid nitrogen^61^. This process enabled complete removal of the glass, enabling visualization of the grid imprint on the exposed Epon surface. The Epon block containing the embedded cells was then coated with a thin layer of gold-palladium (10–20 nm; Edwards S150 sputter coater). Cells of interest were re-localized in the FIB-SEM Helios-600 system (FEI) by matching the fluorescence image to the mirror image obtained by the FIB-SEM. Before working with the sample, a protective layer of platinum of about 30 × 30 × 1 μm was deposited above the expected location of the cell of interest, using the microscope's gas injection system.

The FIB ion beam (30 kV, 20 nA) was used to roughly mill a vertical face along the block, close to this region. In this manner, a wide band of Epon was removed from the front of the region to be imaged. A smaller area within which the final images would be taken was finely polished with a 0.5 nA current. The microscope parameters were then set so that the face was repeatedly milled, and imaged so that serial images were collected through a chosen volume of the block (“slice and view” mode), using voltages of between 2–2.2 kV, with pixel sizes of ~5 nm; a pixel dwell time of 30 μs; and a slice thickness of 10 nm.

The image stack was analyzed by ImageJ. Binning and cropping were applied, in order to make the stack manageable. Images were smoothed and contrast inverted, and a band pass filter applied. Images were then aligned, and misaligned slices were deleted.

Correlations were performed using Amira software (http://www.vsg3d.com/amira/overview), by matching the fluorescence image and the FIB-SEM stack, based on their respective pixel sizes. The FIB-SEM image stack was aligned to match the fluorescence image. Then 3D reconstruction was undertaken, using the voltex tool in the Amira software.

## Author Contributions

O.Y.R. performed all the experiments, and contributed to the design of the experiments and writing of the manuscript. A.W. contributed to Figures 2, 4, and the FIB-SEM imaging. K.R. contributed to Figures 2, 4, and the FIB-SEM imaging. I.S. contributed to Figure 4, and the TEM imaging. A.L. contributed to Figure 6 and the writing of the manuscript. B.G. contributed to the design of the experiments, and the writing of the manuscript.

## Supplementary Material

Supplementary Information Supplementary Movie 1

Supplementary Information Supplementary Movie 2

Supplementary Information Supplementary Movie 3

Supplementary Information Supplementary Movie 4

Supplementary Information Supplementary Movie 5

Supplementary Information Supplementary Movie 6

Supplementary Information Supplementary Movie 7

Supplementary Information Supplementary Movie 8

Supplementary Information Supplementary data
