# Supplementary material for: Mechanical interplay between invadopodia and the nucleus in cultured cancer cells
Source: Sci Rep. 2015 Mar 30;5:9466. doi: 10.1038/srep09466 (PMC4377574; doi:10.1038/srep09466)
Supplement: Supplementary Information — Supplementary data [file srep09466-s9.pdf]

Supplementary data: Mechanical interplay between invadopodia and the nucleus in cultured cancer cells

Or-Yam Revach [1], Allon Weiner [2,4], Katya Rechav[3], Ilana Sabanay [1,3] , Ariel Livne[1], and Benjamin Geiger [1*]

Supplementary Figures

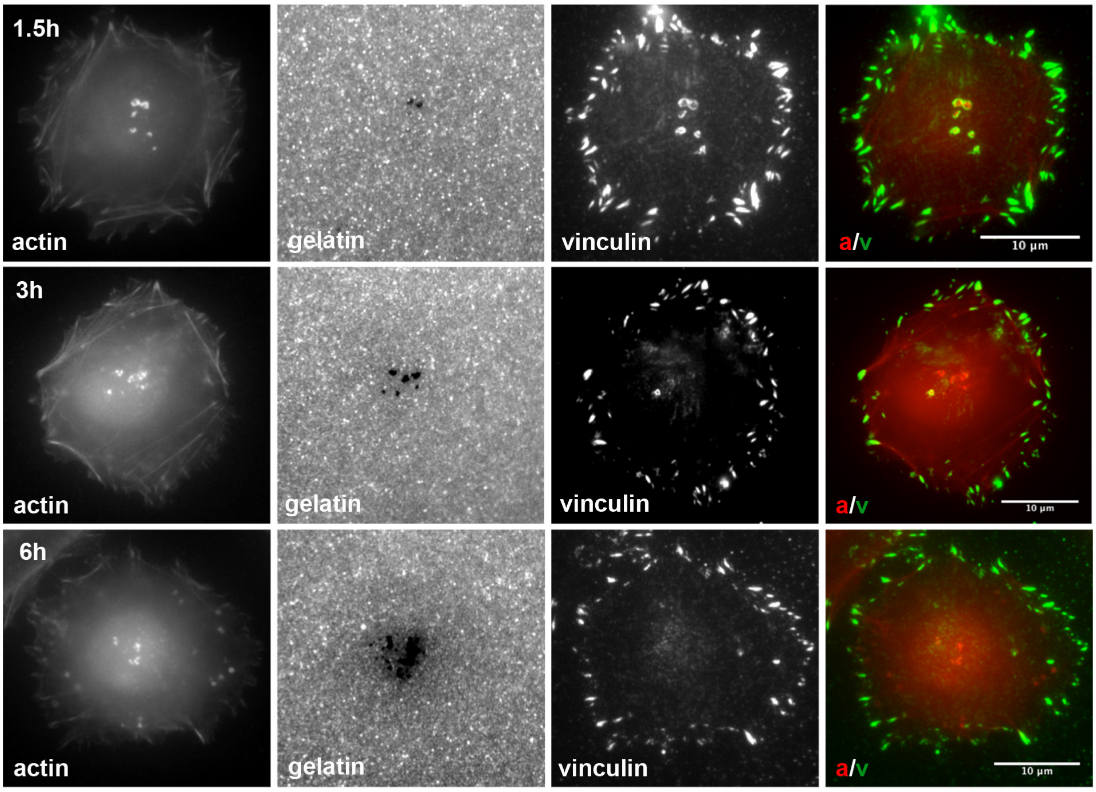

Supplementary Figure 1: Transient association of the adhesion rings with invadopodia.

A375 cells, cultured on 0.2% gelatin-Alexa 488-coated coverslips for 1.5h, 3h, and 6h, then fixed and double-labeled for actin (red) and vinculin (green). The images demonstrate the prominence of the vinculin-containing adhesion ring in "early invadopodia" (1-3 hours after plating), and their absence from more mature invadopodia (~6 h after plating). a/v= actin/vinculin super-position.

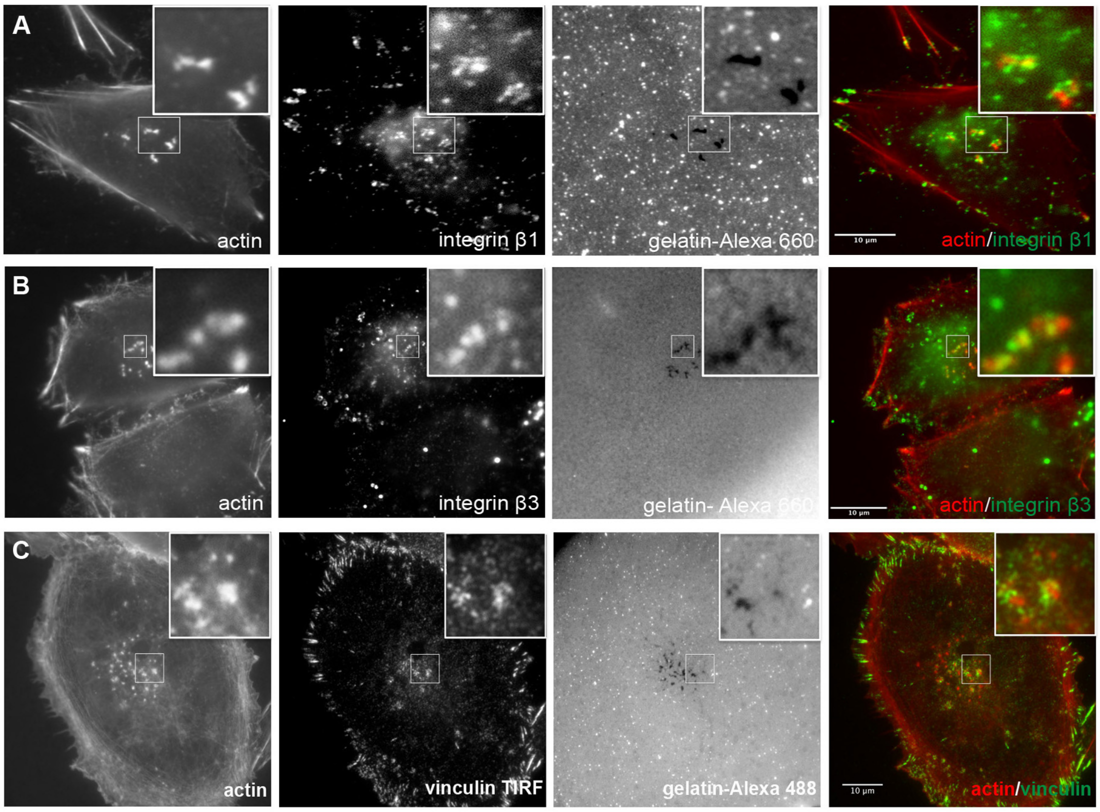

Supplementary Figure 2: Association of integrin- and vinculin-containing adhesion rings with invadopodia.

(A and B): A375 cells, cultured for 2 h on 0.2% gelatin-Alexa 350-coated coverslips. Cells were fixed and co-stained for actin (red) and β 1(A) or β 3 (B) integrins (green). The inserts show an enlarged invadopodia-rich region (marked by a white frame on the full size image). (C) Invadopodia of MDA-231 breast carcinoma cells, displaying adhesion rings, surrounding the F-actin core:MDA-231 breast cancer cells were cultured for 2h on 0.2% gelatin-Alexa 350-coated coverslips. Cells were fixed and co-stained for actin and vinculin. Invadopodia are enlarged in the inserts, as in A and B.

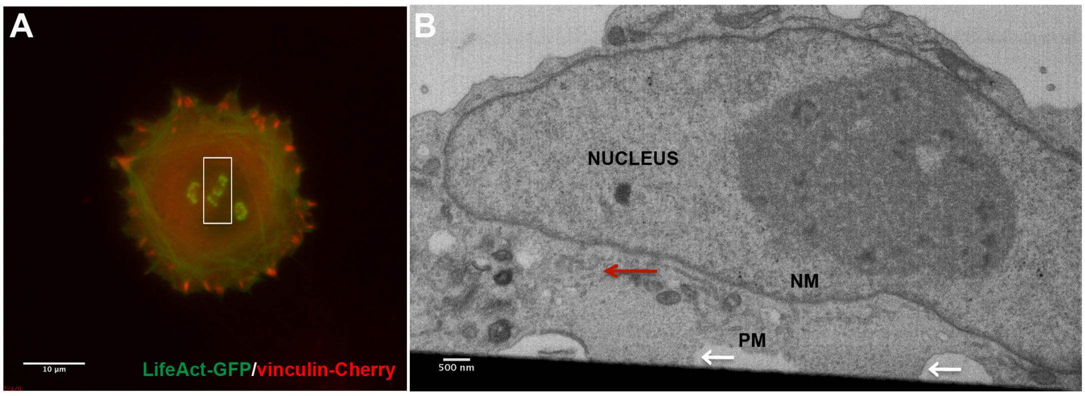

Supplementary Figure 3: An apparent enrichment of endoplasmic reticulum and Golgi membranes around the base of invadopodia.

Correlative fluorescence-FIB-SEM microscopy: (A) A 2D fluorescence image of A375 cells expressing LifeAct-GFP (green) and mCherry-vinculin (red). The white frame delineates a series of invadopodia. (B) A slice of the FIB-SEM stack, showing two invadopodia-based protrusions (marked by white arrows). Endoplasmic reticulum/ Golgi-like membrane vesicles, enriched along the apical region of invadopodia, are marked by the red arrow.

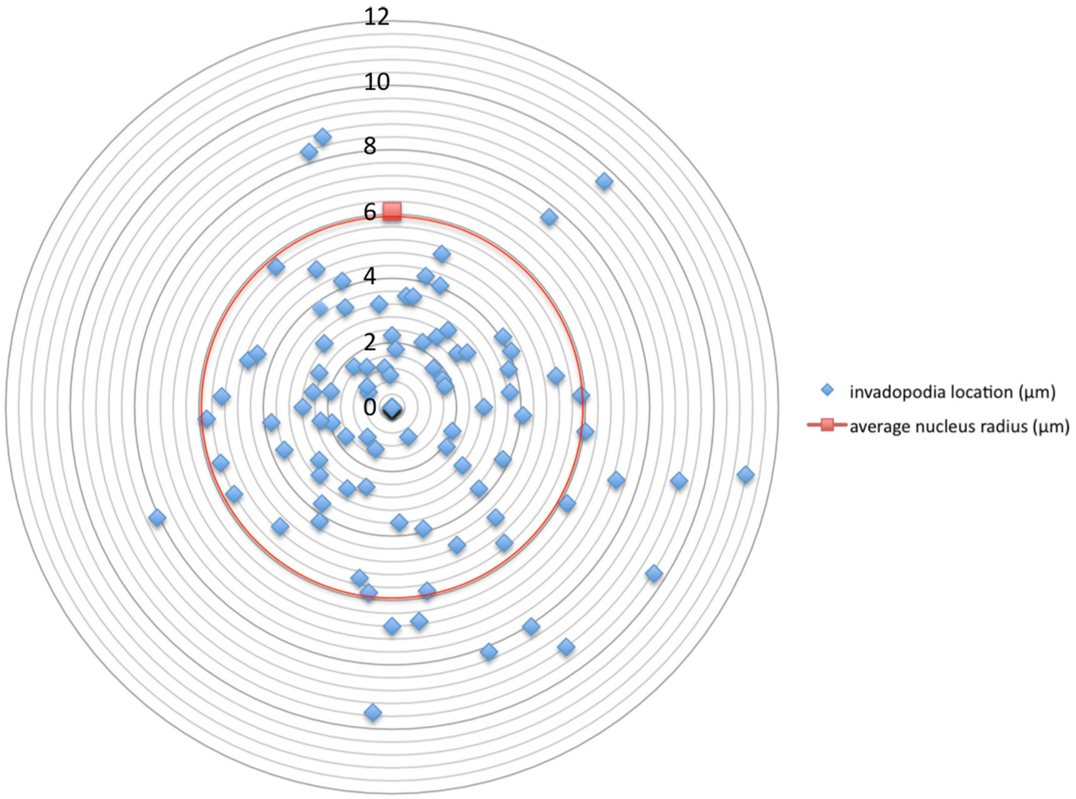

Supplementary Figure 4: Invadopodia in cultured A375 cells are highly enriched in the area subjacent to the nucleus.

A schematic “radar map” view of a cell, depicting the typical subcellular distribution of invadopodia, relative to the cell nucleus. In the cells analyzed (n=100), the average projected radius of the nucleus was 6.09±0.64 µm (marked by the red circle), and the average distance of individual invadopodia (blue diamonds) from the center of the nucleus was 6 µm (range, 0-11.8 µm). Invadopodia distribution in the map shows that over 85% of invadopodia are located “under” the nucleus.

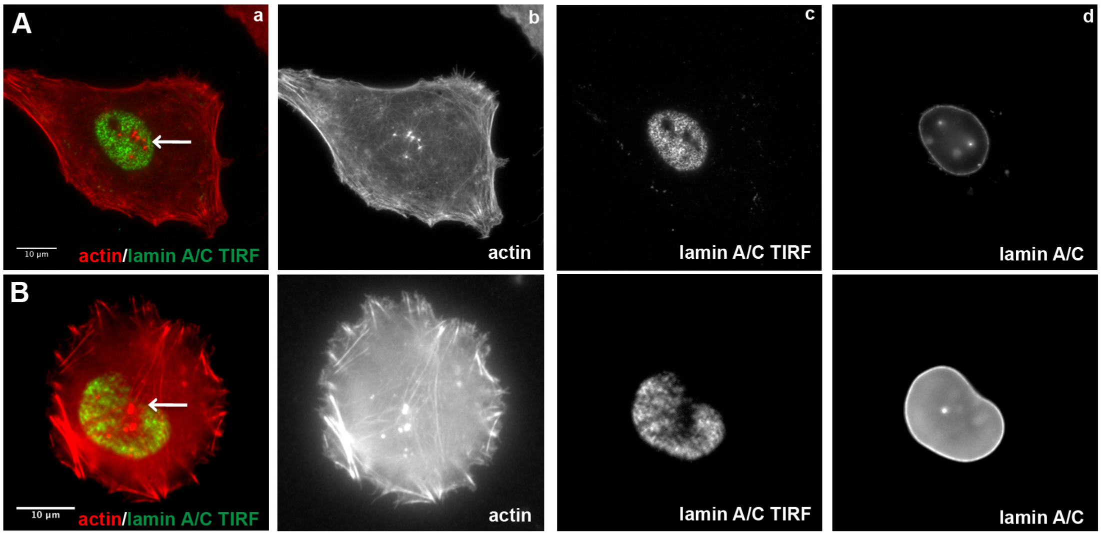

Supplementary Figure 5: Incidence of invadopodia and nuclear indentations in MDA-231 cells, as visualized by TIRF microscopy.

(A) MDA-231 cells were cultured for 2 h on gelatin Alexa 350-coated glass coverslips, then fixed and double-labeled for actin and lamin A/C. (a) Super-imposed image shows invadopodia (labeled by actin) in red, and lamin A/C (using TIRF imaging) in green; invadopodia are noted by white arrows. (b) Actin staining. (c) Lamin A/C staining imaged by TIRF microscopy reveals the nuclear indentation corresponding to the lamin-free area at the ventral aspect of the nucleus. (d) Lamin A/C staining imaged by regular epi-fluorescence microscopy shows that the nuclear lamina is intact. (B) A375 cells were cultured for 2 h on collagen gel coated glass-bottomed dishes, then fixed and double-labeled for actin and lamin A/C. Images from left to right as mentioned above, in a-d.

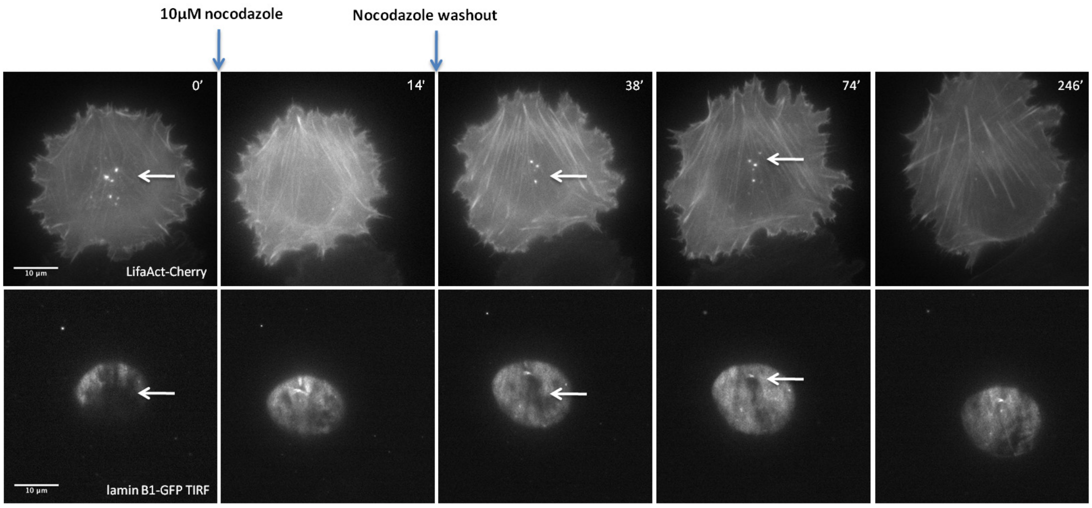

Supplementary Figure 6: Coordinated modulation of invadopodia and nuclear indentation by the addition and withdrawal of the microtubule inhibitor nocodazole.

A375 cells expressing LifeAct-Cherry and Lamin β1-GFP were cultured on gelatin-coated glass-bottomed culture plates for 1-2h, then imaged every 2 min for 6 h, using regular epi-fluorescence microcopy for actin, and TIRF microscopy of lamin β1-GFP, for visualization of the nuclear indentation. Addition of nocodazole-induced disassembly of invadopodia, results in a concomitant disappearance of the nuclear indentation. Washout of the nocodazole from these cells led to the development of new invadopodia, concomitantly with the formation of new nuclear indentations.

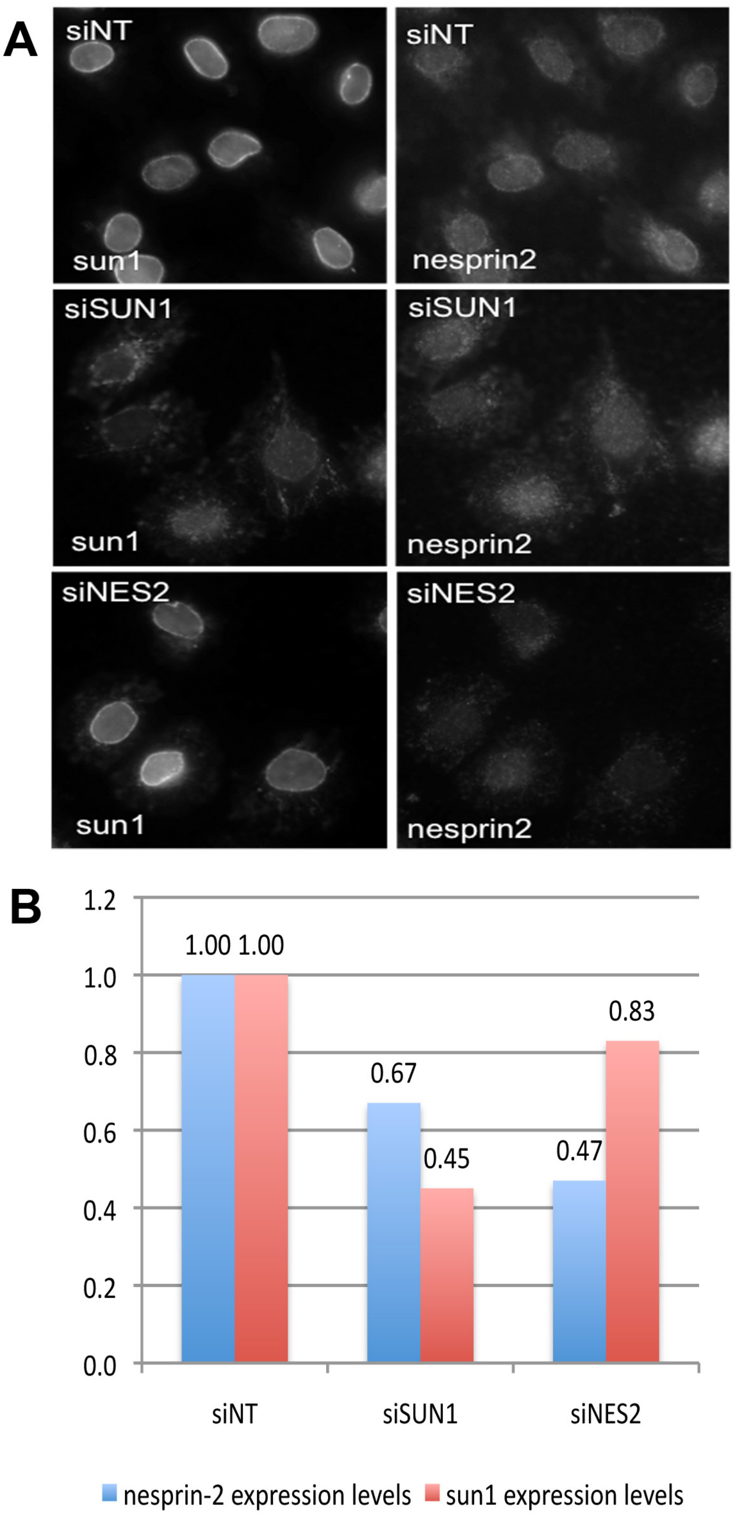

Supplementary Figure 7: The effect of siRNA-mediated knock-down (KD) of LINC complex components on expression levels of SUN2 and Nesprin 2.

A375 cells were transfected with non targeting siRNA (siNT), Nesprin-2 siRNA(siNES2), or sun1 siRNA (siSUN1). After 48h, cells were analyzed for decline of the respective molecules. (A) Immunofluorescence

staining of sun1 and nesprin-2 in control and KD cells. (B) QRT-PCR. Blue bars = nesprin-2 expression levels; red bars = sun1 expression levels. Both genes are normalized to the GAPDH housekeeping gene.

Supplementary Movies:

Supplementary Movie 1: Time-lapse movie of invadopodia cores and zyxin rings.

Supplementary movie corresponding to Fig 1. A375 cells were transfected with Cherry-zyxin and TKS5-GFP constructs. Six hours later, cells were re-plated on gelatin-coated glass-bottomed plates in the presence of 25µm GM6001, and incubated overnight to allow cell spreading. Twenty-four hours post-transfection, the inhibitor was washed away, and cells were imaged at 2 min time intervals for 2h. Invadopodia cores, together with zyxin adhesion rings, formed 45 min after inhibitor withdrawal.

Supplementary Movie 2: Correlative Fluorescence-FIB-SEM characterization of invadopodia.

Supplementary movie corresponding to Fig 2A. A375 cells were transfected with LifeAct-GFP (green) and mCherry-vinculin (red). A 2D fluorescence image is first displayed, followed by a "zoom in" and overlay of the "top-down view" of the FIB-SEM stack. The "top-down view" slice, closest to the matrix, is becoming transparent, revealing the relationships between the FIB-SEM stack and the fluorescence image. The FIB-SEM stack is displayed slice-by-slice, while each invadopod (marked as actin and vinculin in the fluorescence images) is correlated with a membranal protrusion in the EM. Scale bar=10 µm.

Supplementary Movie 3: Invadopodia are surrounded by a web of microtubules.

Supplementary movie corresponding to Fig. 3. A375 cells immune-stained with actin (red), microtubules (green), and lamin A/C (blue). A Z-stack was acquired (Fig. 3) and reconstructed by Imaris® software. A rendering of actin (yellow) and microtubules (blue) shows that microtubules surround invadopodia and are excluded from the core area.

Supplementary Movie 4: Correlative fluorescence-FIB-SEM images show that invadopodia are associated with nuclear indentations.

Supplementary movie corresponding to Fig. 4A. A375 cells were transfected with LifeAct-GFP (green) and mCherry-vinculin (red). A 2D fluorescence image is first displayed, followed by an overlay of the FIB-SEM stack. The "top-down view" of the FIB-SEM is reconstructed, and the correlation of the lower slice to the fluorescence image is shown. Each invadopod (marked as actin and vinculin in the fluorescence image) is correlated with a membranal protrusion in the FIB-SEM stack, as well as with the indented area on the nuclear membrane.

Supplementary Movie 5: 3D reconstruction of the FIB-SEM stack.

Supplementary movie corresponding to Fig. 4B. A 3D reconstruction of A375 cell, using the Voltex module in the Amira software (see Material and Methods). Following the 3D reconstruction, the three indentations in the nuclear membrane are clearly visible.

Supplementary Movie 6: Nuclear indentations as imaged by "dirty TIRF"

Supplementary movie corresponding to Fig. 4E. Lamin A/C was imaged by TIRF microscopy at various angles, using a 660 nm laser (creating evanescence illumination of variable thickness), enabling the acquisition of high resolution Z-axis information and visualization of the entire indentation. For illustration, TIRF angles were reconstructed into 3D surface (yellow) showing the nuclear indentations from a top view and invadopodia actin structures (white) overlay show they are localized inside.

Supplementary Movie 7: Disruption of invadopodia by nocodazole causes flattening of the nuclear indentation

Supplementary movie corresponding to Fig. 5A. A375 cells were transfected with LifeAct-Cherry and Lamin β1-GFP. Cells were imaged every 2 min for 3 h, using standard epi-illumination microcopy for the actin, and TIRF microscopy of lamin β1-GFP, enabling real-time visualization of the nuclear indentation. After 6 min of image recording, nocodazole was added to the cells, leading to invadopodia disassembly, and nuclear indentation flattening.

Supplementary Movie 8: Simultaneous modulation of invadopodia and the associated nuclear indentation by the addition of nocodazole, and its washout

Supplementary movie corresponding to Supplementary Fig. 6. A375 cells were transfected with LifeAct-Cherry and Lamin β1-GFP. Cells were imaged every 2 min for 6 h, using epi-illumination microcopy for actin and TIRF microscopy of lamin β1-GFP, enabling the visualization of the invadopodia-associated actin bundle and the corresponding nuclear indentation. After 4 min, nocodazole was added, leading to disassembly of the invadopodia, and flattening of the nuclear membrane. After disappearance of both the invadopodia and the corresponding indentations, nocodazole was washed out and new invadopodia were formed, concomitantly forming new nuclear indentations. Starting from Frame 63, the nucleus is seen moving away from the associated invadopodia. This nuclear translocation is followed by destabilization of the invadopodia, and their apparent disassembly.
